# sc2DAT: workflow for targeting tumor subpopulations of single cells

**DOI:** 10.1093/bioadv/vbaf237

**Published:** 2025-09-26

**Authors:** Giacomo B Marino, Anna I Byrd, Nasheath Ahmed, Daniel J B Clarke, Avi Ma’ayan

**Affiliations:** Department of Pharmacological Sciences, Mount Sinai Center for Bioinformatics, Icahn School of Medicine at Mount Sinai, New York, NY 10029, United States; Department of Pharmacological Sciences, Mount Sinai Center for Bioinformatics, Icahn School of Medicine at Mount Sinai, New York, NY 10029, United States; Department of Pharmacological Sciences, Mount Sinai Center for Bioinformatics, Icahn School of Medicine at Mount Sinai, New York, NY 10029, United States; Department of Pharmacological Sciences, Mount Sinai Center for Bioinformatics, Icahn School of Medicine at Mount Sinai, New York, NY 10029, United States; Department of Pharmacological Sciences, Mount Sinai Center for Bioinformatics, Icahn School of Medicine at Mount Sinai, New York, NY 10029, United States

## Abstract

**Summary:**

The rapid increase in volume, diversity, and quality of single-cell omics profiling opens new opportunities for drug and target discovery. While there are already many workflows developed for analysis and visualization of data collected with single-cell RNA-seq, few workflows output ranked drugs and targets specific for subpopulation of single cells. Here, we present the single cells to drugs and targets (sc2DAT) workflow, a web-based software application for predicting cell surface targets and therapeutic compounds tailored to target-specific cell types automatically identified from scRNA-seq and bulk RNA-seq datasets. sc2DAT can be used to develop hypotheses about selectively eliminating malignant subpopulation of cells in cancer, or reprogram disease tissues toward a healthier phenotype using compounds from the LINCS L1000 dataset. Such compounds are hypothesized to either reverse or mimic the direction of changes in gene expression signatures, restoring the subpopulation of cells towards a healthier phenotype.

**Availability and implementation:**

sc2DAT is available from: https://sc2dat.maayanlab.cloud; the source code is available from: https://github.com/MaayanLab/sc2DAT.

## 1 Introduction

Normal tissue and cell type atlases such as Genotype-Tissue Expression (GTEx) (GTEx Consortium 2013), the Human BioMolecular Atlas Program (HuBMAP) (HuBMAP Consortium 2019), and the Human Cell Atlas (HCA) (Regev *et al.* 2017) can serve as a reference for identifying genes that are only highly expressed in disease while lowly expressed in normal tissues and cell types. Such genes, which may be only expressed during certain stages in development, or under extreme physiological conditions, can become ideal drug targets. This is because these genes are only highly expressed in disease cells, minimizing the impact of drugs, and other targeted therapeutics, on normal healthy cells and tissues. Prior work has shown that by comparing tumor samples profiled with RNA-seq to normal expression tissue and cell atlases, we can reveal selectively expressed genes suitable for targeted therapies. For instance, Bosse *et al.* identified GPC2 as a tumor-specific cell-surface protein in neuroblastoma by contrasting the tumor and GTEx transcriptomes (Bosse *et al.* 2017). This approach has been applied systematically to reveal previously undiscovered targets for 12 pediatric cancers (Orentas et al., 2012). We used a similar approach to rank cell-surface targets for 69 cancer subtypes identified for 10 cancers profiled by the Clinical Proteomic Tumor Analysis Consortium (CPTAC) (Deng *et al.* 2024).

To democratize similar approaches, several bioinformatics applications have emerged to support target and drug discovery directly from transcriptomics (Table 1, available as supplementary data at *Bioinformatics Advances* online). For example, the Open Cancer TherApeutic Discovery (OCTAD) platform (Zeng *et al.* 2021) enables users to utilize gene expression profiling of tumors from cancer patients, normal tissues, including those from GTEx, alongside drug perturbation datasets to enable virtual compound screening. Recently, we have developed TargetRanger (Marino *et al.* 2023) and Multiomics2Targets (Deng *et al.* 2024) two bioinformatics platforms that focus on identifying cell surface proteins uniquely upregulated in tumors and absent across normal tissues sourced from ARCHS4 (Lachmann *et al.* 2018), GTEx (GTEx Consortium 2013), and Tabula Sapiens (Tabula Sapiens Consortium *et al.* 2022). Similarly, QSurface (Hong *et al.* 2018) is a desktop application that can be used to query cell-surface proteins across cancer subtypes. However, QSurface is limited in scope because it only considers a predefined set of membrane targets. Focusing on mutations, QSurface lacks integration with normal tissue data. Within this domain, a large-scale computational screen identified combinations of antigens that improve tumor discrimination for CAR-T cell therapies. 60 million combinations across 33 tumor types and 34 normal tissues were scanned to find dual and triple antigen strategies (Dannenfelser *et al.* 2020).

At the same time, Connectivity Mapping efforts have profiled the response of cell lines to thousands of chemical perturbations (Keenan *et al.* 2019). For example, the Library of Integrated Network-based Cellular Signatures (LINCS) Common Fund NIH program profiled the response of over 200 human cell lines to thousands of small molecules and CRISPR knockout (KO) perturbations with the L1000 transcriptomics assay (Keenan *et al.* 2018). Such resources can be used as reference databases for identifying perturbations that reverse the direction of change in gene expression signatures (reversers) or mimic it (mimickers) when queried with gene expression signatures profiled in different contexts (Marino *et al.* 2025). However, both the cell surface target identification and Connectivity Mapping approaches are not yet widely applied to prioritize drugs and targets for single-cell transcriptomics expression datasets.

Single-cell RNA sequencing (scRNA-seq) is becoming increasingly accessible and cost-effective, revealing cell type-specific gene expression patterns. For example, in cancer, tumors are composed of diverse subpopulations of cells, while in many other complex diseases, specific cell types exhibit distinct dysregulation. Thus, targeting these subpopulations of cells more precisely may be more effective and safer than treating all cells in bulk tumors or diseased tissues indiscriminately. This can be achieved by aggregating reads for identified cell types from scRNA-seq data (pseudobulking). Additionally, with the use of deconvolution algorithms, and a scRNA-seq reference atlas, cell type fractions as well as bulk-like cell type-specific expression vectors can be inferred from bulk RNA-seq data.

Some efforts have been made to specifically target subpopulations of tumor cells (Table 1, available as supplementary data at *Bioinformatics Advances* online). For instance, the Beyondcell method ranks therapeutic compounds tailored to specific tumor cell subpopulations (Fustero-Torre *et al.* 2021). In a related effort, TIMEDB assembled ∼40 000 transcriptomics samples of profiled tumors from 43 cancer types, estimating their cell-type composition via deconvolution analysis and visualization (Wang *et al.* 2023b). Resources such as TIMEDB can accelerate the identification of tumor subpopulations of cells that could be targeted. For example, it is established that some immune cells in the tumor microenvironment are pro-tumorigenic, while other immune cells are pro-inflammatory, naturally assisting the patient to eliminate the tumor. Hence, specifically targeting the pro-tumorigenic cells in the tumor microenvironment can assist in overcoming resistance to targeted and untargeted cancer therapies (Wang *et al.* 2023a).

Here we present single cells to Drugs and Targets (sc2DAT), a workflow that can be used to identify, and target, specific subpopulations of cell types. Starting with either bulk or single-cell RNA-sequencing samples collected from a tumor, or a diseased tissue, sc2DAT ranks cell-surface targets, CRISPR knockouts (KOs), and chemical perturbations that are hypothesized to either kill the diseased cells or push their expression profile toward a healthier phenotype. Overall, the sc2DAT workflow and web server application are designed for experimental biologists who aim to discover and prioritize personalized treatments for complex human diseases.

## 2 Methods

### 2.1 scRNA-seq normalization and integration

The scRNA-seq data normalization follows the standard Seruat vignette (Butler *et al.* 2018). First, genes expressed in <3 cells, and cells that express <500 genes, are filtered out using scanpy (Wolf *et al.* 2018). Then, doublets, an artifact that arises when multiple cells are mistakenly assigned the same barcode, are removed using scrublet (Wolock *et al.* 2019); and then cells with >5% mitochondrial expressed genes are removed. The remaining cell and gene matrix is normalized such that the sum of the gene counts in each cell is 10 000. The resulting expression vectors are log transformed, and the top 2000 genes with the most variance are retained for dimensionality reduction and visualization. Dimensionality reduction and visualization is performed with Uniform Manifold Approximation Projection (UMAP) (McInnes *et al.* 2018). If two conditions of scRNA profiles are compared, then integration is performed using Harmony (Korsunsky *et al.* 2019), generating a harmonized principal component analysis (PCA) representation of the single-cell profiles used for visualization and downstream analysis.

### 2.2 Normalization and deconvolution (bulk RNA-seq)

Bulk RNA-seq data is quantile and log2 normalized. Dimensionality reduction and visualization with UMAP (McInnes *et al.* 2018) is performed on the normalized data. Deconvolution of the bulk RNA-seq samples is performed with BayesPrism (Chu *et al.* 2022) through a fast implementation in R called InstaPrism (Hu and Chikina 2024). Single-cell reference backgrounds are pre-computed from a variety of sources including Tabula Sapiens (Tabula Sapiens Consortium *et al.* 2022) and Tabula Muris (Tabula Muris Consortium 2020). InstaPrism provides both cell type fractions as well as cell-type-specific expression inferred for each bulk RNA-seq sample. To prioritize cell types for downstream analysis, we rank the cell types by the most altered cell type fractions by comparing the control (normal) with the perturbation (disease) conditions.

### 2.3 Cell type annotation (scRNA-seq)

Cells in the combined conditions are clustered using the Leiden algorithm (Traag *et al.* 2019) and visualized as a 2D UMAP (McInnes *et al.* 2018). Canonical cell type markers are sourced from PanglaoDB (Franzén *et al.* 2019) and are used for enrichment analysis with the decoupleR package. Enrichment for each cell type is determined using the t-test for overestimated variance. The top three enriched cell types for each cluster are selected for visualization. Each cluster is annotated with the most enriched cell type.

### 2.4 Pseudobulking by cell type (scRNA-seq)

To maintain the statistical power of the downstream differential expression analysis, we generate three bulk-like replicates for each cell type using random sampling. Each determined or provided cell type is “pseudobulked” by summing a random selection of half of that cell type population three times. A random seed is used to ensure reproducibility. This creates three unique representative samples. These vectors of expression are further normalized by aligning them to each background atlas' distribution for target identification.

### 2.5 Cell surface target identification

To identify genes that are highly expressed in the target cells while lowly expressed across normal human cell types and tissues we utilize TargetRanger (Marino *et al.* 2023). TargetRanger compares the inputted samples with processed RNA-seq data from several atlases containing healthy tissue and cell type gene expression profiles, namely, ARCHS4 (Lachmann *et al.* 2018), GTEx (GTEx Consortium 2013), and Tabula Sapiens (Tabula Sapiens Consortium *et al.* 2022) for human, and ARCHS4 (Lachmann *et al.* 2018) and Tabula Muris (Tabula Muris Consortium 2020) for mouse. The simulated cell type-specific bulk samples are compared to the healthy reference backgrounds, and then significantly up-regulated targets are identified. Users have the option to validate the identified targets by cross-referencing them with DepMap by choosing cell lines derived from a relevant tumor type (Tsherniak *et al.* 2017). DepMap provides scores that quantify the cell viability effect of knocking-down single genes in cancer cell lines. The gene effect scores are averaged across the selected cancer cell lines. Gene-knockout effect scores range from 0 to −1, representing the median effect of non-essential and essential genes, respectively (Meyers *et al.* 2017). This provides a measure of potential efficacy for each identified target from TargetRanger.

### 2.6 LINCS L1000 reversers

To identify compounds and gene knockouts (KOs) most likely to push the disease cell types to a more healthy phenotype, we query SigCom LINCS (Evangelista *et al.* 2022). SigCom LINCS is a web-based search engine that serves over 1.5 million gene expression signatures processed, analyzed, and visualized from the LINCS L1000 dataset (Subramanian *et al.* 2017). To query SigCom LINCS, up and down gene sets are computed for each cell type by comparing the control and perturbation cell type-specific samples with the Welch’s *t*-test and filtering for genes with a *P*-value of <.01. The extracted gene sets are submitted for analysis with SigCom LINCS. SigCom LINCS provides rapid signature similarity search for mimickers and reversers given the sets of up and down genes. The top 10 reverser compounds and CRISPR KOs reversers are visualized with bar charts and provided as tables by sc2DAT.

## 3 Results

**Figure 1.**
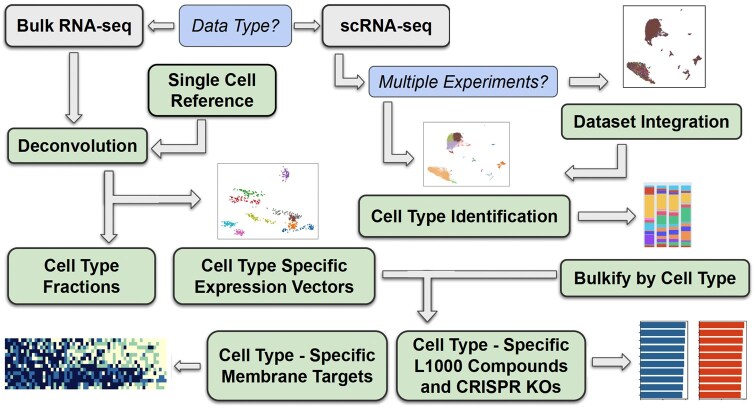
The sc2DAT workflow. The workflow starts with either a single-cell profile or bulk RNA-seq dataset that has control and perturbation conditions along with a tissue-specific single-cell reference. Then sc2DAT utilizes the LINCS L1000 dataset with SigCom LINCS to identify compounds most likely to push the diseased cell types to a healthy phenotype, as well as TargetRanger for identifying cell-type-specific membrane targets for targeted cell removal as determined by TargetRanger.

The sc2DAT pipeline enables users to identify cell type-specific targets as well as reverser and mimicker compounds and CRISPR KOs for either a bulk or single-cell RNA-seq datasets. A single-cell reference is required for the bulk RNA-seq upload, and single-cell expression profiles from multiple conditions can be optionally uploaded for scRNA-seq data analysis (Fig. 1). For bulk RNA-seq data, we include 20 precomputed single-cell references covering major human and mouse tissues (Table 2, available as supplementary data at *Bioinformatics Advances* online). To demonstrate the utility of the workflow, we provide two use cases, one that begins with bulk and the other with single-cell RNA-seq.

### 3.1 Cell type-specific targets for lung cancer

The example use case of using sc2DAT to analyze bulk RNA-seq begins with visualizing the bulk RNA-seq expression profiles collected from the lung adenocarcinoma (LUAD) cohort collected by CPTAC3, including the corresponding normal adjacent tissues (NATs) (Li *et al.* 2023) (Fig. 1A, available as supplementary data at *Bioinformatics Advances* online). Next, using BayesPrism (Chu *et al.* 2022) and InstaPrism (Hu and Chikina 2024) with the Lung Cancer Atlas dataset (Salcher *et al.* 2022) as the single-cell reference, cell type fractions and cell-type-specific bulk expression vectors are computed and visualized as stacked bar charts and UMAP plots (McInnes *et al.* 2018) (Fig. 1B–D, available as supplementary data at *Bioinformatics Advances* online). Using the two conditions, tumor and NAT, the cell types are compared by their change in proportion. For example, we observe a large change in the fraction of the stromal cell subpopulation (Fig. 1B, available as supplementary data at *Bioinformatics Advances* online). For this cell type subpopulation, cell-surface targets are identified. These targets are highly expressed in the tumor stromal cells, while lowly expressed across most healthy human tissues and cell types using the TargetRanger algorithm with the ARCHS4 (Lachmann *et al.* 2018), GTEx (GTEx Consortium 2013), and Tabula Sapiens (Tabula Sapiens Consortium *et al.* 2022) backgrounds (Fig. 1E, available as supplementary data at *Bioinformatics Advances* online). Additionally, for each of these targets, gene knockdown effect scores from DepMap are plotted (Tsherniak *et al.* 2017) based on their effect on non-small cell lung cancer cell (NSCLC) cell lines (Fig. 1F, available as supplementary data at *Bioinformatics Advances* online). For this use case, the sc2DAT workflow was used to identify cell surface targets that may be suitable as immunotherapies for the development of antibody-drug conjugates (ADCs) and chimeric antigen receptor (CAR) T-cell therapies. One such target, KCNS3, has been already identified as a therapeutic target for multiple cancers including lung cancer (Zúñiga *et al.* 2022). Additionally, PIEZO2 exhibits a highly negative gene effect score in the lung cancer cell lines, suggesting that targeting it directly may lead to cell death in the subpopulation of tumor cell types, without the need of a conjugate drug. Using SigCom LINCS (Evangelista *et al.* 2022) we also identified reverser compounds and gene KOs that may push tumor cell subpopulations toward the healthy phenotype. For stromal cells, which are known to be altered in lung cancer, and are associated with cancer progression (Kamimura *et al.* 2024), a top reverser predicted drug is menadione, also known as vitamin K3 (Fig. 1G, available as supplementary data at *Bioinformatics Advances* online). Menadione has been previously identified as a promising anti-cancer drug in conjunction with other treatments such as chemotherapy in NSCLC (Soltanian and Sheikhbahaei 2021). Additionally, for endothelial cells, which play multiple roles in lung adenocarcinoma progression, including inhibition of immune responses (Bantikassegn *et al.* 2015), celecoxib was identified as a top reverser compound (Fig. 1H, available as supplementary data at *Bioinformatics Advances* online). Celecoxib is a selective COX-2 inhibitor, commonly used as an anti-inflammatory agent but has more recently been studied in the context of treatment for multiple cancer types including colon, breast, and lung (Tołoczko-Iwaniuk *et al.* 2019). Specifically, celecoxib has been shown to induce apoptosis in the NSCLC cell lines A549 and H460 (Kim *et al.* 2017). For tumor endothelial cells, the CYP3A4 CRISPR KO was identified as a highly significant reverser. In fact, CYP3A4 expression levels are extremely high in some lung adenocarcinoma tissues. High levels of CYP3A4 expression are associated with poor survival (He *et al.* 2020).

### 3.2 Cell type-specific targets for acute kidney injury

The single-cell RNA-seq use case utilized data from a study examining repair pathways in acute kidney injury (AKI) (Gerhardt *et al.* 2021). AKI can be caused by ischemia, sepsis, or toxins and can lead to chronic kidney disease (CKD). It is known to damage proximal tubule cells, activating a poorly understood self-repair process. When this repair fails, CKD can develop. To better understand the repair process, a mild-to-moderate ischemia–reperfusion injury (IRI) mouse model was utilized to mimic clinical scenarios such as kidney transplantation. The sc2DAT pipeline starts by visualizing the single-nucleus RNA sequencing (snRNA-seq) of genetically labeled injured kidney cells at 7 and 28 days post-IRI (Fig. 2A, available as supplementary data at *Bioinformatics Advances* online) with a UMAP plot, coloring single cells by their experimental group or batch. Then, the Leiden algorithm (Traag *et al.* 2019) is utilized to cluster the cells and the cell type for each cluster is determined using the overrepresentation of marker genes from PanglaoDB (Franzén *et al.* 2019) (Fig. 2B, available as supplementary data at *Bioinformatics Advances* online). UMAP is then used to visualize the identified cell types in each cluster (Fig. 2C, available as supplementary data at *Bioinformatics Advances* online). Next, TargetRanger is applied with mouse-specific backgrounds computed from ARCHS4 (Lachmann *et al.* 2018) and Tabula Muris (Tabula Muris Consortium 2020) to identify cell-type-specific targets across the diseased cell populations (Fig. 2D, available as supplementary data at *Bioinformatics Advances* online). To specifically remove proximal tubule cells which may exhibit senescent properties, Tspan5 was identified as a potential target. Tspan5 is known to interact with Adam10 to induce inflammation and potentially contribute to AKI (Reyat *et al.* 2017). Additionally, Itga1 was identified as another potential target for proximal tubule cells. Itga1 plays a crucial role in cell migration and adhesion and has been identified as both biomarker for therapeutic resistance and metastasis in pancreatic cancer (Grenier *et al.* 2024) as well as a major positive regulator of kidney fibrosis, where it was identified as a potential therapeutic target (Gharibi *et al.* 2017). The TargetRanger algorithm in this context can identify selective targets for disease cell types, as well as reveal underlying biology with some cell-surface targets significantly up-regulated compared to healthy tissues and cell types across many of cell types in the AKI model. Following target identification, sc2DAT queries SigCom LINCS (Evangelista *et al.* 2022) to rank reverser compounds and CRISPR KOs for each cell type in the diseased profile. The second-ranked CRISPR KO for proximal tubule cells is SLC6A13 (Fig. 2E, available as supplementary data at *Bioinformatics Advances* online). SLC6A13 depletion in a mouse model was reported to reduce rental cellular senescence, inflammation, and fibrosis (Oe and Vallon 2023). Additionally, a top-ranked compound for reversing diseased proximal tubule expression was LY-215490, which is a competitive and selective AMPA receptor antagonist studied for its neuroprotective effects in animal models of stroke and ischemia (Gill and Lodge 1994). It is possible that the compound could also exhibit protective effects from ischemia in the kidney model. Another vulnerable cell type in AKI is the podocyte. A top ranked CRISPR KO reverser for the podocyte cell type population is PRDX4 (Fig. 1F, available as supplementary data at *Bioinformatics Advances* online). PRDX4 has been shown to interact with the dopamine D5 receptor to reduce inflammation and oxidative stress in the kidney and in the mouse model its knockout led to greater oxidative damage (Iuchi *et al.* 2009). Overall, the two use cases demonstrate how the sc2DAT application and workflow can be applied in different disease scenarios that collect bulk or single-cell RNA-sequencing data from human and mouse tissues.

### 3.3 Additional use cases

We provide additional use cases that demonstrate how sc2DAT can be used to identify cell surface targets for three distinct conditions: subtypes of dendritic cells from spleen whole-blood samples (GSE137710; Brown *et al.* 2019), pancreatic adenocarcinoma (Savage *et al.* 2024), and Crohn’s disease (Kong *et al.* 2023). An overview of each use case is provided in Table 3, available as supplementary data at *Bioinformatics Advances* online.

### 3.4 Time requirements and resource limitations

The sc2DAT workflow can be computationally intensive and has finite memory access. This is potentially limiting for large single-cell datasets. To provide insight into the capacity of sc2DAT, we tested the site with three single-cell and two bulk RNA-seq datasets of increasing size. The size and time requirements of each use case are reported in Table 3, available as supplementary data at *Bioinformatics Advances* online. Additionally, we identified that doublet prediction is a time limiting step in the single-cell workflow. Thus, we now provide it as an opt-in feature for users of large datasets. We recommend that users who wish to perform doublet prediction on large datasets run sc2DAT locally.

## Supplementary Material

vbaf237_Supplementary_Data
