# Supplementary material for: sc2DAT: workflow for targeting tumor subpopulations of single cells
Source: Bioinform Adv. 2025 Sep 26;5(1):vbaf237. doi: 10.1093/bioadv/vbaf237 (PMC12512136; doi:10.1093/bioadv/vbaf237)
Supplement: vbaf237_Supplementary_Data [file vbaf237_supplementary_data.pdf]

Supporting Online Materials

for

sc2DAT: Workflow for Targeting Tumor Subpopulations of Single Cells

Giacomo B. Marino[1], Anna I. Byrd[1], Nasheath Ahmed[1], Daniel J. B. Clarke[1], Avi Ma'ayan[1,*]

[1]Department of Pharmacological Sciences, Department of Artificial Intelligence and Human Health, Mount Sinai Center for Bioinformatics, Icahn School of Medicine at Mount Sinai, New York, NY 10029, USA.

*To whom correspondence should be addressed.

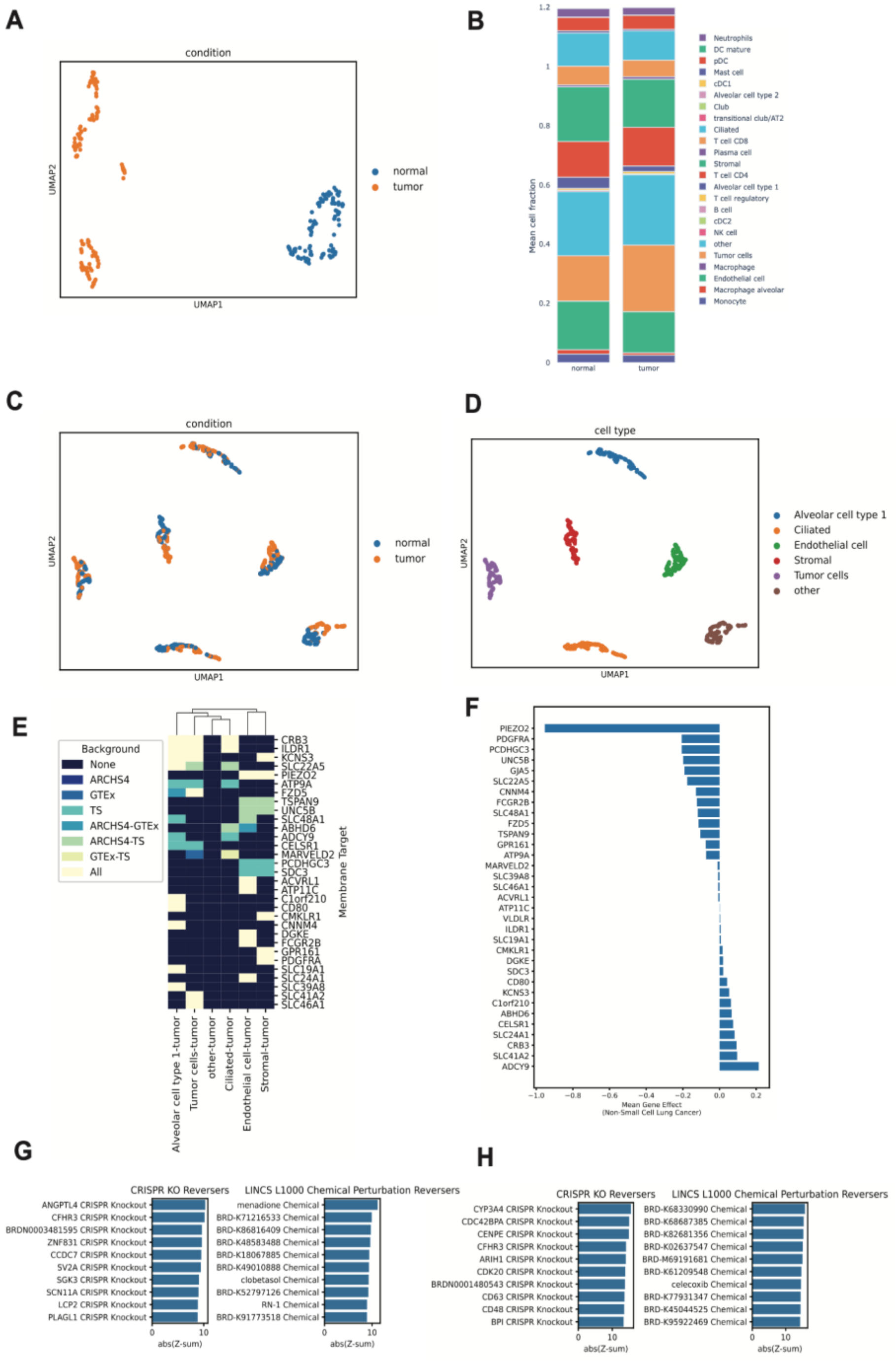

Figure S1. Identifying targets and therapeutic compounds for specific cell types in the CPTAC3 LUAD cohort. **A.** UMAP visualization of quantile and log-normalized bulk RNA-seq gene expression data colored by tumor or normal tissue for the CPTAC3 LUAD cohort; **B** Stacked bar chart of cell type fractions per condition computed using the selected cell type reference and InstaPrism; **C.** UMAP visualization of cell type specific bulk expression vectors for most altered cell type populations colored by experimental condition; **D.** UMAP visualization of cell type specific bulk expression vectors for most altered cell type populations colored by cell-type; **E.** Membrane targets identified for cell types within a single tumor; **F.** DepMap gene effect scores following knockout in non-small cell lung cancer cell lines for the identified targets. **G.** LINCS L1000 CRISPR KOs and chemical perturbation reversers for the tumor stromal cells subpopulation; **H.** LINCS L1000 CRISPR KOs and chemical perturbation reversers for the endothelial cells identified as a subpopulation in the tumor.

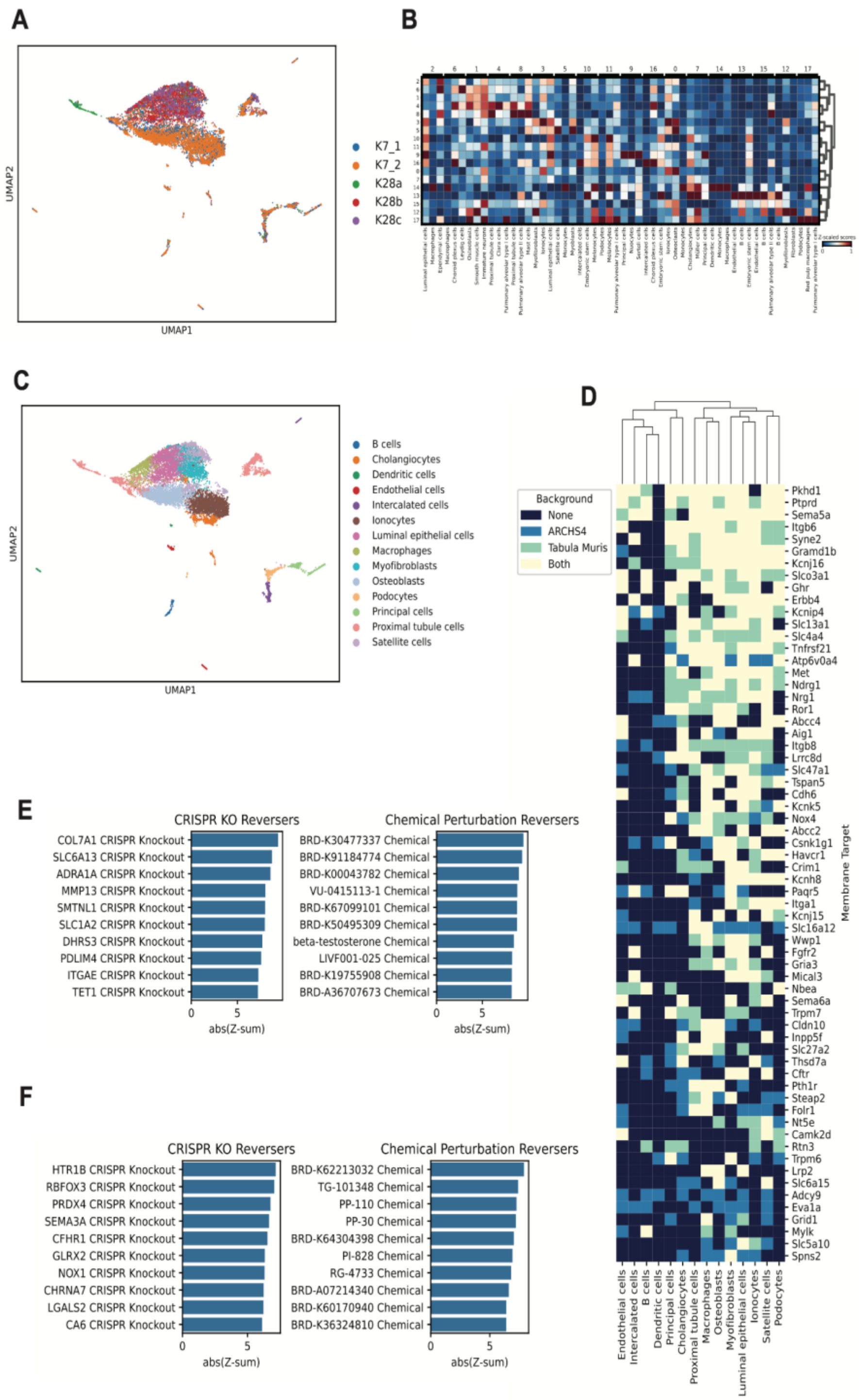

Figure S2. Identifying compounds and targets for dysregulated cell types in acute kidney injury model in mice. **A.** UMAP visualization of harmonized single cell datasets, colored by groups where K7 is an early model at 7 days post injury and K28 is 28 days post injury; **B.** Matrix visualization of scRNA-seq leiden cluster cell type enrichments; **C.** UMAP visualization colored by predicted cell types; **D.** Membrane Targets for perturbed cell types, filtered by genes upregulated in control cell types compared to healthy background atlases; **E.** LINCS L1000 CRISPR KO and chemical perturbation reversers for identified proximal tubule cells; **F.** LINCS L1000 CRISPR KO and chemical perturbation reversers identified for podocytes.

Tool	PMID	A	B	C	D	E	F	G	H	I	J
sc2DAT	--	✓	✓	✓	✓	✓	✓	✓	✓	✓	✓
Multiomics 2 Targets	39127042	✓	✓	✓	✓	✓	✓	x	✓	x	x
Target Ranger	37166966	✓	✓	✓	✓	x	✓	✓	✓	x	x
OCTAD	33361798	✓	✓	x	✓	✓	✓	✓	x	x	x
QSurface	29560830	✓	x	✓	✓	✓	✓	x	x	✓	x
ImmunoTar	39932005	✓	x	✓	✓	x	✓	x	✓	x	x
SurfaceGenie	32053146	✓	✓	✓	✓	x	x	x	✓	x	x
ASGARD	36813801	✓	x	x	x	x	✓	✓	x	✓	x
BeyondCell	34911571	✓	x	✓	x	x	x	✓	✓	✓	x
TimeDB	36399488	✓	✓	✓	x	x	✓	x	✓	x	x

Table S1. Comparison of sc2DAT against tools that recommend subpopulation-specific targets or drugs. **A** Open source, **B:** Web interface, **C:** Interactive visualization of results, **D:** Identifies disease-specific cell surface targets, **E:** Performs differential expression between control vs. case samples, **F:** Compatible with bulk RNA-seq data, **G:** Compatible with single-cell RNA-seq data, **H:** Accepts user data as input, **I:** Identifies chemical perturbations that are hypothesized to kill diseased cells, or revert cells to healthy phenotype, **J:** Identifies CRISPR KO experiments that are hypothesized to kill diseased cells.

Tissue	Source	Species
Pancreas	Tabula Sapiens	Human
Adipose Tissue	Tabula Sapiens	Human
Adipose Tissue	Tabula Muris	Mouse
Liver	Tabula Sapiens	Human
Blood	Tabula Sapiens	Human
Kidney	Tabula Sapiens	Human
Kidney	Lake et al. 2023	Human
Brain (non-myeloid)	Tabula Muris	Mouse
Heart	Tabula Sapiens	Human
Heart	Tabula Muris	Mouse
Muscle	Tabula Sapiens	Human
Muscle	Tabula Muris	Mouse
Skin	Tabula Sapiens	Human
Lung	Tabula Sapiens	Human
Lung	Tabula Muris	Mouse
Spleen	Tabula Sapiens	Human
Spleen	Tabula Muris	Mouse
Prostate	Tabula Sapiens	Human
Lung (Cancer Atlas)	Salcher et al. 2022 (LuCA)	Human

Table S2. Single-cell reference backgrounds available from the sc2DAT pipeline.

RNA-seq Type	Sample Description	Data source	Original Publication	Largest File Size (unzipped)	Time to complete (seconds)
Bulk	Tumor and NAT from patients with PDAC	Linked Omics (PMID:29136207)	PMID:38917788	43.9 MB	822.371
Bulk	Tumor and NAT from patients with LUAD	CPTAC3 (PMID:36720220)	PMID:36720220	63 MB	353.721
Single cell	Epithelial cells of the colon in patients with and without Crohn's Disease	Single Cell Portal (PMID:37502904)	PMID:36720220	2.23 GB	456.098*
Single cell	Spleen whole-blood sample	Gene Expression Omnibus (PMID:23193258)	PMID:31668803	146.7 MB	210.956
Single cell	Recurrent Kidney Injury	Gene Expression Omnibus (PMID:23193258)	PMID:34183416	566.4 MB	378.405

Table S3. Summary of additional use cases. Time requirements were determined via using Google Chrome version 138.0.7204.184 on a 13-inch MacBook Pro (2023) with an M2 chip and 16GB of random-access memory, running MacOS 14.6.1.
